# Ethnobiology and Ethnopharmacology of *Lepidium meyenii* (Maca), a Plant from the Peruvian Highlands

**DOI:** 10.1155/2012/193496

**Published:** 2011-10-02

**Authors:** Gustavo F. Gonzales

**Affiliations:** Department of Biological and Physiological Sciences, Faculty of Sciences and Philosophy and High Altitude Research Institute, Universidad Peruana Cayetano Heredia, Honorio Delgado 430, Lima 31, Peru

## Abstract

*Lepidium meyenii* (maca) is a Peruvian plant of the Brassicaceae family cultivated for more than 2000 years, which grows exclusively in the central Andes between 4000 and 4500 m altitude. Maca is used as a food supplement and also for its medicinal properties described traditionally. Since the 90s of the XX century, an increasing interest in products from maca has been observed in many parts of the world. In the last decade, exportation of maca from Peru has increased from 1,415,000 USD in 2001 to USD 6,170,000 USD in 2010. Experimental scientific evidence showed that maca has nutritional, energizer, and fertility-enhancer properties, and it acts on sexual dysfunctions, osteoporosis, benign prostatic hyperplasia, memory and learning, and protects skin against ultraviolet radiation. Clinical trials showed efficacy of maca on sexual dysfunctions as well as increasing sperm count and motility. Maca is a plant with great potential as an adaptogen and appears to be promising as a nutraceutical in the prevention of several diseases.

## 1. Introduction

*Lepidium meyenii *Walpers (maca) is a Peruvian plant growing over 4000 m with high potential for bioprospecting [1]. Maca has been used for centuries in the Andes for nutrition and to enhance fertility in humans and animals [1, 2]. The demand for food particularly with benefits for health is high, but it will increase over the future years. Then, the search of plants with these potentials is of interest.

This plant belongs to the brassica (mustard) family and Lepidium genus [1]. The most relevant plants related to *Lepidium meyenii* are rapeseed, mustard, turnip, black mustard, cabbage, garden cress, and water cress. Lepidium constitutes one of the largest genera in the Brassicaceae family. The species from North America and Europe has been extensively studied, and the *Lepidium meyenii* from the Andean region has recently been studied profusely because of the great health benefits [3–5]. Maca grows at a habitat of intense cold, extremely intense sunlight, and strong winds. Maca is used as a food supplement and for its presumed medicinal properties [3].

The Peruvian native population in the central Andes use the hypocotyls after it has been naturally dried and in amounts >20 g/d. There are no reports of adverse reactions after consuming *Lepidium meyenii *in food [4]. However, natives from the highlands of Peru recommend that maca be boiled before its consumption because fresh maca may have adverse effects on health [5]. The effects of fresh maca on health have not been scientifically assessed yet. Preparations from the maca hypocotyls were reported to be of benefit for health [3–5]. 

The hypothesis that maca may be effective in improving health status, particularly reproductive function, is supported by several lines of evidence. Historical aspects and biological properties of maca, gathered from experimental and clinical studies on this species, reveal the importance of this plant as nutraceutical food, and that maca was adapted to conditions as harsh as observed at high altitude [2, 3, 5–7]. The aims of this review are to summarize and assess the evidence from experimental and clinical studies for or against the effectiveness of maca in the improvement of different functions.

## 2. History and Tradition

Maca has been cultivated in the Peruvian central Andes, in the former Chinchaycocha (Plateau of Bombón); present-day: Carhuamayo, Junin, and Óndores in the Junin Plateau close to Cerro de Pasco [2]. Maca was probably domesticated in San Blas, Junin (present day: Ondores) some 1,300–2,000 years ago.

The first written description about maca (as a root without identification of the botanical or popular name) was published in 1553, in which Cieza de Leon, a chronicler of the Spaniard conquest of Peru noted that in the Peruvian highlands, particularly in the province of Bombón (Chinchaycocha; present day: Junin) the natives used certain roots for maintenance [6]. The roots, he was referring to were maca. 

Father Cobo [2] was the first to describe the name of maca and its properties in 1653. He stated that this plant grows in the harshest and coldest areas of the province of Chinchaycocha where no other plant for man's sustenance could be grown. Cobo also referred to the use of maca for fertility. In the 18th century, Ruiz referred to the fertility-enhancing properties of maca and also its stimulant effect [7]. I believe stimulant effect could be related to energizer effect or an effect on mood or well-being. 

**Figure 1:**
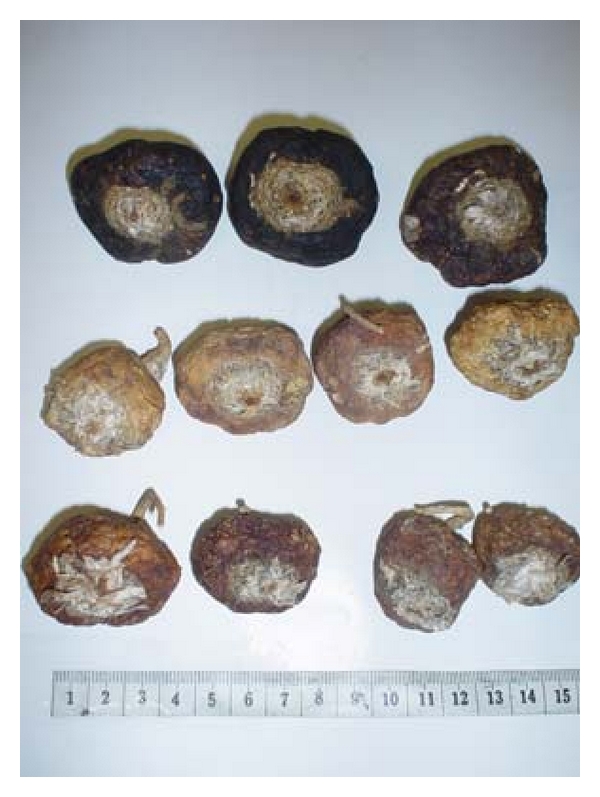
Dried hypocotyls of naturally dried black (upper), yellow (middle), and red (bottom) maca.

Traditionally, after being harvested maca is dried naturally and can thus be stored for many years [5]. The dried hypocotyls are hard as stone (Figure 1). After being naturally dried maca hypocotyls can be eating. Before eaten, the hypocotyls need to be boiled in water to obtain a soft product which can be consumed as juice, the most frequent form of use [4]. 

The boiling process seems to increase active metabolites. In fact, increased temperature affects the availability of several secondary metabolites in plants sometimes increasing some metabolites and in others a reduction in metabolites is observed. In maca, one of the important constituents is glucosinolates. These compounds are sensitive to heating [8]. Other metabolites, however, are increased after heating. For instance, heating decreases the activity of epithiospecifier protein and increases formation of sulforaphane, a derivative of isothiocyanates and glucosinolates, in broccoli [9]. 

After 2, 15, and 30 min of heating at 88°C, the vitamin C content of raw tomato drops significantly. Yet, the content of translycopene per gram of tomato increases [10]. Moreover, antioxidant activity also increases after heating tomatoes [10].

## 3. Ethnobiology

Maca is characterized by an overground and an underground part. The overground part is small and flat in appearance. This seems to be the result of an adaptation process to prevent the impact of strong winds. The underground part is the hypocotyl-root axis.

The principal and the edible part of the plant is a radish-like tuber that constitutes the hypocotyl and the root of the plant. This hypocotyl-root axis is 10–14 cm long and 3–5 cm wide and constitutes the storage organ storing a high content of water. After natural drying, the hypocotyls are dramatically reduced in size to about 2–8 cm in diameter (Figure 1). The average weight of the dried hypocotyls may vary considerably. For instance, in our experience, we found a range of weight between 7.64 and 23.88 g in the Peruvian central Andes.

There are many types of maca that can be characterized by the color of their hypocotyls. In Carhuamayo, Junin, in the Peruvian highlands, 13 colors of maca have been described, ranging from white to black [11]. Recently, it has been demonstrated that different types of maca (according to its color) have different biological properties [16, 20, 35].

## 4. Chemistry

Primary metabolites correspond to the nutritional component of the hypocotyls, and the secondary metabolites to compound with biological and medicinal properties.

### 4.1. Primary Metabolites

The dried hypocotyls of maca are approximately 13–16% protein, and are rich in essential amino acids. Fresh hypocotyls contain 80% water and have high amounts of iron and calcium (see [5]). A more complete description of the composition of dry maca shows [12] 10.2% proteins, 59% carbohydrates, 2.2% lipids, and 8.5% of fibre. Free fatty acids are also present in maca, the most abundant being linoleic, palmitic, and oleic acids. Saturated fatty acids represent 40.1% whereas unsaturated fatty acids are present at 52.7%. 

Maca contains amino acids (mg/g protein) like leucine (91.0 mg), arginine (99.4 mg), phenylalanine (55.3 mg), lysine (54.3 mg), glycin (68.30 mg), alanine (63.1 mg), valine (79.3 mg), isoleucine (47.4 mg), glutamic acid (156.5 mg), serine (50.4 mg), and aspartic acid (91.7 mg). Other amino acids present but in less proportion are histidine (21.9 mg), threonine (33.1 mg), tyrosine (30.6 mg), methionine (28.0 mg), hydroxyproline (26 mg), proline (0.5 mg), and sarcosine (0.70 mg). Minerals reportedly found in maca were iron (16.6 mg/100 g dry matter), calcium (150 mg/100 g dry matter), copper (5.9 mg/100 g dry matter), zinc (3.8 mg/100 g dry matter), and potassium (2050 mg/100 g dry matter) among others (see [5]).

### 4.2. Secondary Metabolites

Maca contains several secondary metabolites [5]. The secondary metabolites macaridine, macaene, macamides, and maca alkaloids are only found in this plant [13]. Macaenes are unsaturated fatty acids [13]. Other compounds include sterols as beta-sitosterol, campesterol, and stigmasterol. 

Different glucosinolates as the aromatic glucosinolate glucotropaeolin have been described within maca. Benzyl glucosinolate has been suggested as chemical marker for maca biological activity. However, this has been discarded since glucosinolates may easily metabolize to isothiocyanates and these in other smaller metabolites [14]. 

Benzyl glucosinolate is also present in another Peruvian plant named mashua (*Tropaeolum tuberosum*). This plant, however, has opposed effects to maca since administration to male rats reduced sperm count [15] in contrast with the known effect of maca increasing sperm count [16]. 

It has been observed that maca batches from different producers significantly vary in the amount of macaene, macamides, sterols, and glucosinolates [17–19]. In 2005 appeared the first publication indicating that different maca color types have different properties [20]. More recently, it has been found that maca colors associate with variations in concentrations of distinct bioactive metabolites [19, 21]. These compounds individually or acting in synergy may be acting favoring the reported biological properties from maca.

The differences in proportion of secondary metabolites between maca colors may explain different biological properties described for maca.

## 5. Ethnopharmacology of Maca

### 5.1. Experimental Studies

Since 2000 to this date, several studies have been reported on biological or pharmacological effect of maca on experimental animals. The results have been consolidated in Table 1. 

The process of preparation of maca is important to obtain adequate biological effects. Traditionally maca is boiled or extracted in alcohol before it is consumed [4]. In experimental studies, aqueous extract of maca is only effective after boiling pulverized maca hypocotyls in water. 

The greatest effect on spermatogenesis was observed with the ethyl acetate fraction of the hydroalcoholic extract of black maca [42]. Extract after boiling (Aqueous extract) has similar effect of hydroalcoholic extract of maca [14]. In fact, the effect of maca on benign prostatic hyperplasia (BPH) seems to be related with the content of benzyl glucosinolate. Both, aqueous and hydroalcoholic extract of red maca, to a similar extent, reduced the prostate weight in rats with prostatic hyperplasia induced by testosterone enanthate (TE) [14].

## 6. Experimental Studies on Reproduction

### 6.1. Male Reproduction

#### 6.1.1. Sexual Function

Treatments of experimental animals with pulverized maca hypocotyls in doses of 15, 25, 75, and 100 mg/kg and the assessment of sexual behavior at 1, 7, 15, and 21 days of treatment yielded different results [22, 24]. The first study found increased sexual behavior of males at treatment days 1 and 15 [22] whereas the second study did not find changes in male sexual behavior at treatment days 1 or 21 [24]. Macaenes and macamides have been reported as novel compounds in maca [13] and probably responsible to improve sexual behavior [13], although this needs to be further demonstrated.

#### 6.1.2. Sperm Function

Maca has been found to increase sperm count in normal rats and in pathological conditions produced by exposure to high altitude [42], lead acetate injections [43], and malathion [44]. Maca also increases sperm motility [16]. Black maca and in minor proportion yellow maca are the varieties responsible to increase sperm count and sperm motility whereas red maca had no effect [16].

#### 6.1.3. Prostate Function

Testosterone enanthate (TE) administered to mice [34] and rats [14, 20, 45] induced prostatic hyperplasia. Red maca administered with TE for 21 and 42 days to male rats or mice prevented the prostatic hyperplasia. Yellow maca had intermediate effects and black had not effect on prostate size. In fact, red maca reduced prostate weight in a dose-response manner without any changes in testosterone levels and seminal vesicle weight [14, 45]. Regarding the secondary metabolites involved in the effect of red maca on prostate size, when different doses of benzylglucosinolates in red maca extracts were assessed, a dose-dependent reduction in prostate weight was observed, suggesting that these compounds may be responsible for the biological effect of red maca [14]. However, other secondary metabolites presented in red maca could be also responsible for the effect on prostate size. In fact, other authors found that polyphenols could inhibit prostate size [46, 47]. Recently, it was suggested that polyphenols in red maca may be related to the reduction in prostate size [34].

Prostate zinc levels were increased by TE administration, an experimental model to induce prostatic hyperplasia. Red maca was able to reduce zinc levels in TE-treated rats. Although red maca was able to reverse the effect of TE administration in prostate weight and zinc levels, no effect was observed in seminal vesicle weight, another androgen-dependent organ [20]. Finasteride, the standard pharmacological treatment for prostatic benign hyperplasia, which inhibits the activity of the enzyme 5 alpha reductase which in turn blocks the conversion of testosterone to dihydrotestosterone, was able to reduce both prostate and seminal vesicle weights but did not completely reduce zinc levels in prostate (unpublished data). Our results may possibly suggest that red maca and finasteride could have different mechanisms of action. In fact, previous studies showed that red maca specifically affects prostate size without altering testosterone or estradiol levels either in mice or in rats with prostatic hyperplasia induced by TE [14, 20, 45]. Also, it has been published that maca has no effect on androgen receptor [48, 49]. The latter supports the hypothesis that red maca effect is at a postandrogen receptor action level [14] or that RM exerts an inhibitory effect at a level postdihydrotestosterone conversion [45].

The finding that maca reduces benign prostatic hyperplasia (BPH) is a contribution of science since no traditional description refer to this effect. This is comprehensible since BPH occurs since 50 years of age, and before century XX, expectancy of life was below 50 years.

#### 6.1.4. Serum Hormone

Testosterone controls sexual desire and spermatogenesis. However, the effect of maca on these physiological processes does not seem to be regulated by changes in serum testosterone or intratesticular testosterone levels. However, the mechanism is not yet known [48]. Thus, further chemical and molecular research is required to identify which of the many components of maca accounts for the effects observed.

### 6.2. Female Reproduction

Serum estradiol levels were not affected in different studies which used mice [50], rats [16, 45], or humans [51]. Moreover, with an *in vitro* assay in our laboratory, we could not show that maca has a proliferative effect on MCF-7 cells [Vaisberg and Gonzales, unpublished observations].

Maca extract has been demonstrated to improve the number of offsprings in mice [33]. This effect seems to be due to an effect favoring survival of embryos. This has also been suggested in rainbow trouts [39, 40]. Recently, in our laboratory it has been demonstrated that extract of red maca is more effective to improve quality of embryos in mice (unpublished data). 

Extracts of red and black maca have protective effects on bone architecture in ovariectomized rats without showing estrogenic effects on uterine weight [28]. This finding may suggest the possibility to study effect of extracts of maca for treatment of women with osteoporosis.

## 7. Memory and Learning

Although no traditional descriptions have been found about effect of maca on learning and memory, actually natives in the central Peruvian Andes ascribe to the use of maca in children improves school performance. They do not exactly know which variety of maca has better effect on memory and learning. Experimental studies have shown that black variety of maca has beneficial effects on learning and memory in experimental animal models. Black maca improved learning and memory in ovariectomized mice [35, 37] and in scopolamine-induced memory impairment in mice [36].

Three varieties have been studied (black, red, and yellow maca) and black maca was the only on showing significant biological effects [35]. Studies have been performed using hydroalcoholic extracts of maca or boiled aqueous extract of maca. Both were similarly effective in improving memory and learning [35–37]. Black maca (0.5 and 2.0 g/kg) decreased brain malondialdehyde (MDA) levels marker of oxidative stress and acetylcholinesterase (Ache) levels in ovariectomized mice whereas no differences were observed in monoamine oxidase (MAO) levels [37]. Black maca seems to improve experimental memory impairment induced by ovariectomy, orchidectomy, scopolamine, and alcohol due in part to by its antioxidant and Ache inhibitory activities.

In summary, different evidences suggest that maca, particularly black maca, improves learning and memory.

## 8. Studies in Humans

Interest in maca has in increased worldwide during the last 10 years. This increased interest in maca has also been accompanied by some concern about safety. Piacente et al. (2002) [52] described the presence of (1R,3S)-1-methyl-1,2,3,4-tetrahydro-*β*-carboline-3-carboxylic acid (MTCA) in maca hypocotyls. On this finding, the authors made some generalizations about the action of MTCA suggesting that it can be toxic. These affirmations have motivated the French Agency for Sanitary Security (AFFSA) issued an opinion about the risk for the health of the consumer using the pulverized roots of maca [53]. However, MTCA also occurs on fruits like oranges and grapefruit and fruit juices [54], which are frequently used because of their favorable properties on health. MTCA has been described on the fermented garlic extract [55, 56], and its concentration increases with time, in turn increasing its antioxidant activity. Moreover, MTCA is detected in several foods, and in some, in concentrations relatively high (greater than the ones found by Piacente in maca) suggesting that claims are overestimated. 

In a recent paper, several arguments indicate that MTCA in maca is safe [57]. In addition, maca is not mutagenic but it contains several beneficial compounds, some of which have anticarcinogenic properties [5, 58]. The consumption of maca must not generate concern, taking in account that, as mentioned in the French alert [53], it has not been reported any toxicity in the case of maca traditional consumption that requires a boiling process. MTCA is a natural constituent of several plants and on consumption of such plants there is no toxicity found. This suggests that as a multicomponent it may lose its adversity as drug action.

Furthermore, a recent study was designed to investigate health status in a population from the Peruvian central Andes (Carhuamayo, 4100 m) which traditionally consumes maca and compared it with a population from the same place which does not consume maca. The study, based on a survey, assessed maca consumption, sociodemographic aspects, health status, and fractures in men and women aged 35–75 years old. In a subsample were assessed the hepatic and kidney functions and hemoglobin values. From the sample studied, 80% of the population consumed maca. 85% of them consume maca for a nutritional purpose. 

**Figure 2:**
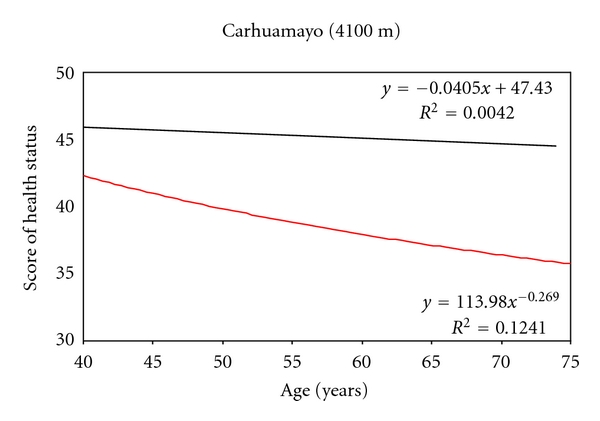
Score of health status from men and women residents of Carhuamayo, Junin at 4100 m in the Peruvian Central Andes. Upper line: population consuming extracts of maca. Bottom line: population not consuming maca; Source: [4].

Maca is used since childhood and mainly after hypocotyls it is naturally dried. The consumption is mainly as juices, and the variety that they consume is a mixture of different colors of the hypocotyls. Maca consumption is associated with higher score in health status (Figure 2), lower rate of fractures, and lower scores of signs and symptoms of chronic mountain sickness. In addition, maca consumption is associated with low body mass index and low systolic blood pressure. 

Hepatic and kidney function, lipidic profile, and glycemia were normal in the population consuming maca. In summary, this study demonstrated in a population traditionally using maca that consumption of this food is safe [4].

## 9. Maca and Sexual Function

Sexual dysfunctions are highly prevalent in our society worldwide, and the occurrence of sexual dysfunctions increases directly with age for both men and women [59]. They occur in 20–30% of men and 40–45% of women according to 18 descriptive epidemiological studies from around the world [60]. 

Most sexual problems relate to sexual desire (interest in sex) in both females and males and male erectile dysfunction (ED) [60]. Interest in medicinal plants to treat sexual dysfunctions has increased in the last 20 years [61]. 

Maca has been described to improve sexual behavior in experimental animals [13, 22, 23], although conflictive results may be observed [24]. Traditionally maca has been referred to as a plant to improve fertility [2] and as an energizer [3]. In a randomized study we were unable to demonstrate effect of maca on penile erection in apparently healthy adult men after 12 weeks of treatment with gelatinized maca compared with results using placebo [Gonzales, unpublished data].

Recently, a systematic review has been performed on effect of maca on sexual function in humans [62]. In this review, according to the authors only four randomized clinical trials (RCT) met all the inclusion criteria [49, 63–65]. 

According to the review, two RCTs suggested a significant positive effect of maca on sexual dysfunction or sexual desire in healthy menopausal women [49] or healthy adult men [63], respectively, while the other RCT according to the reviewers failed to show any effects in healthy cyclists. However, analyzing results from such study, authors showed that maca extract significantly improved the self-rated sexual desire score compared to the baseline test (*P* = 0.01), and compared to the placebo trial after supplementation (*P* = 0.03) [64]. The effect in this study was as early as 14 days of treatment which is significantly shorter that that showed with gelatinized maca in which effects were observed after 8 weeks of treatment. 

A further RCT assessed the effects of maca in patients with mild erectile dysfunction using the International Index of Erectile Dysfunction-5 and showed significant effects on subjective perception of general and sexual well-being [65]. 

A study was not included in the systematic review because no placebo effect was assessed [66]. In such study, maca was administered in two doses (1.5 g/day and 3–0 g/day) to patients with selective-serotonin reuptake inhibitor-(SSRI-)induced sexual dysfunction. The Arizona Sexual Experience Scale (ASEX) and the Massachusetts General Hospital Sexual Function Questionnaire (MGH-SFQ) were used to measure sexual dysfunction. 

Subjects on 3.0 g/day maca had a significant improvement in ASEX (from 22.8 ± 3.8 to 16.9 ± 6.2; *z* = −2.20, *P* = 0.028) and in MGH-SFQ scores (from 24.1 ± 1.9 to 17.0 ± 5.7; *z* = −2.39, *P* = 0.017), but subjects on 1.5 g/day maca did not. Libido improved significantly (*P* < 0.05) based on ASEX item number 1, but not by dosing groups. Maca was well tolerated [66]. 

Although evidence suggests an effect of maca on sexual desire and mild erectile dysfunction data also revealed that maca extract seems to have better effect [64] than gelatinized maca [63] and that of maca flour. The difference seems to be due to the fact that extract allow the concentration the secondary metabolites. 

In summary, there is evidence that maca may improve sexual desire but is inconclusive an effect on erectile function.

## 10. Maca and Sperm Function

In a study in 9 apparently healthy men who had received maca for 4 months showed an increase in seminal volume, sperm count, and sperm motility [67] (Table 2). Serum hormone levels (LH, FSH, prolactin, estradiol, and testosterone) in men were not affected by treatment with maca [51, 67]. 

Maca powder and maca extract were unable to activate androgen receptor-mediated transcription in prostate cancer cell lines [48] or in a yeast-based hormone-dependent reporter assay [49].

In summary, experimental and one clinical studies suggest that consumption of maca is associated with an increase in sperm count.

## 11. Maca as an Energizer

Maca has been shown to reduce scores in depression and anxiety inventories [49, 66]. A self-perception survey showed that maca acted as energizer compared with placebo in apparently healthy men [3]. 

Maca extract administration for 14 days significantly improved 40 km cycling time performance compared to the baseline test (**P** = 0.01), but not compared to the placebo trial after supplementation (**P** > 0.05). 

In summary, scientific evidences suggest that maca may be an energizer.

## 12. Maca and Metabolic Syndrome

One study has been reported on effects of maca alone or combined with another supplements in patients with metabolic syndrome. The randomized placebo-controlled 90-day study assessed the effects of maca and yacon in combination with silymarin on plasma and lipoprotein lipids, serum glucose, and safety parameters in patients suffering from the metabolic syndrome. 

No adverse effects were found in volunteers using silymarin (0.8 g/day), silymarin + yacon (0.8 + 2.4 g/day), and silymarin + maca (0.6 + 0.2 g/day). A moderate AST level and diastolic blood pressure increase was found in volunteers using maca (0.6 g/day) [68]. 

However, a randomized clinical trial in healthy men showed that gelatinized maca reduced systolic and diastolic blood pressure after 12 weeks of treatment [3]. Moreover, maca significantly inhibited the hypertension relevant angiotensin I-converting enzyme (ACE) *in vitro* [69]. 

In a population traditionally consuming maca, systolic blood pressure was lower than in those not consuming maca [4]. Similarly, AST levels were similar in those consuming and those not consuming maca [4]. 

Maca contains high amounts of potassium [5]. Potassium is an important nutrient to reduce risk of hypertension [70] and as a primary metabolite may be useful in patients with hypertension. In addition other secondary metabolites may be also be active to reduce blood pressure [69].

## 13. Maca and Osteoarthritis

In a randomized double-blind study on 95 patients with osteoarthritis, a combination of *Uncaria guianensis *(cat's claw; 300 mg) and maca (1,500 mg) was administered twice a day for 8 weeks and compared with a treatment with glucosamine sulfate. Both treatments substantially improved pain, stiffness, and functioning in the patients [71]. However, as the study did not include a placebo control group, glucosamine effects remain unclear.

## 14. Toxicity

Maca has been used for centuries in the Central Andes of Peru, and no toxic effects have been reported if it was consumed after boiling [5]. Previous review data on *in vivo* and *in vitro* studies with maca indicate that its use is safe [5]. Further evidence shows that aqueous and methanolic extracts of maca do not display *in vitro* hepatotoxicity [72]. Moreover, freeze-dried aqueous extract of maca (1 g/kg BW) in mice did not reveal any toxic effect on the normal development of preimplanted mouse embryos [73].

Results in rats show that different types of maca (black, red, and yellow) have no acute toxicity at ≤17 g of dried hypocotyls/kg BW. Rats treated chronically for 84 days with 1 g/Kg BW showed no side effects and a histological picture of liver similar to that observed in controls [74]. As usual doses in rats are 1-2 g/Kg BW, it is suggested that maca is safe. Human consumption of ≤1 g/kg per day is considered safe, as well. However, as referred above in a study in patients with metabolic syndrome the administration of maca at a dose of 0.6 g/day for 90 days resulted in a moderate elevation of AST and diastolic arterial pressure [68]. This has not been confirmed in other studies [3, 4]. Data on population of 600 subjects in the Peruvian central Andes showed that maca consumption was safety and that health status was improved [4].

## 15. Final Comments

**Figure 3:**
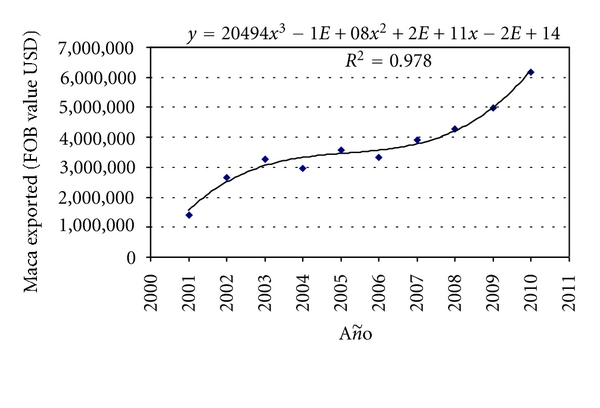
Maca exported from Peru in the last 10 years. Data are in FOB values (USD).

Consumption of Maca worldwide has significantly increased during the last 10 years. This is depicted in Figure 3 which presents data on maca export from Peru, the only country producing maca. During 2010, Peru exported maca for a value of 6,179,011.8 USD, 4.36-times higher than value exported during 2001.

Clearly, further research is required to address the mechanisms of actions and the active principles of this plant. However, available data suggest that maca has several important biological properties, and scientific evidence of these properties could be important for farmers, dealers, and consumers. Furthermore, it is necessary to demonstrate the biological effects of specific secondary metabolites of maca and their actions when added as a mixture. 

Maca is a plant with great potential as an adaptogen and appears to be promising as a nutraceutical in the prevention of several diseases. Scientific evidence showed effects on sexual behavior, fertility, mood, memory, osteoporosis, metabolism, and the treatment of some tumor entities. However, the active principles behind each effect are still unknown. Macamides have been described as novel compounds of maca that have not been found in any other plant species so far [13]. It is suggested that this lipid fraction of maca may be responsible for the increase in sexual behavior [13, 23]. Studies on testicular function, spermatogenesis, fertility, mood, memory, and prostatic hyperplasia [16, 35, 42, 75] were performed with aqueous extracts that contain only trace amounts of macamides [17]. This suggests that compounds other than macamides are responsible for these activities.

## Figures and Tables

**Table 1:** Properties for maca after *in vivo* administration in experimental animals.

Species	Property	Source
Rats	Increase sperm count and sperm motility	[16]
	Increase male sexual behavior	[13, 22, 23]
	Small effect on rat male sexual behavior	[24]
	Nutritional	[25]
	Antistress	[26, 27]
	Prevent testosterone-induced prostatic hyperplasia	[20]
	Reversed osteoporosis	[28, 29]
	Neuroprotective effects	[30]
	Protects against UV radiation	[31]
	antioxidant status, lipid, and glucose metabolism	[32]

Mice	Increase male sexual behavior	[13]
	Increase embryo survival	[33]
	Prevent testosterone-induced prostatic hyperplasia	[34]
	Increase number of offsprings	[33]
	Improve memory and learning	[35–37]

Guinea pigs	Increase number of offsprings	[38]

Fish	Nutritional	[39, 40]
	Increase embryo survival	[39]

Bulls	Improve sperm quantity and quality	[41]
	Unaffected mating behavior	

**Table 2:** Semen variables before and 4 month after maca treatment.

Semen variable	Before maca *N* = 9	After maca *N* = 9	*P* value
Volume (mL)	2.23 ± 0.28	2.91 ± 0.28	<0.05
pH	7.47 ± 0.09	7.44 ± 0.07	NS
Sperm count (10^6^/mL)	67.06 ± 18.61	90.33 ± 20.46	NS
Total sperm count (10^6^/mL)	140.95 ± 31.05	259.29 ± 68.17	<0.05
Motile sperm count (10^6^/mL)	87.72 ± 19.87	183.16 ± 47.84	<0.05
Sperm motility grade a (%)	29.00 ± 5.44	33.65 ± 3.05	NS
Sperm motility grade a + b (%)	62.11 ± 3.64	71.02 ± 2.86	<0.05
Normal sperm morphology (%)	75.50 ± 2.02	76.90 ± 1.23	NS

Data are mean ± standard error of the mean. *N* = number of subjects, NS: not significant, source: [67].
